# Anthropometry and Body Composition of Adolescents in Cracow, Poland

**DOI:** 10.1371/journal.pone.0122274

**Published:** 2015-03-27

**Authors:** Wiesława Klimek-Piotrowska, Mateusz Koziej, Mateusz K. Hołda, Katarzyna Piątek, Karolina Wszołek, Anna Tyszka, Elizabeth Kmiotek, Mateusz Pliczko, Aleksandra Śliwińska, Klaudia Krauss, Marcin Miszczyk, Jerzy Walocha

**Affiliations:** Department of Anatomy, Collegium Medicum, Jagiellonian University, Cracow, Poland; University of Bremen, GERMANY

## Abstract

**Background and Objective:**

The aim of the present study was to determine the level of adiposity and obesity in Polish adolescents and compare the results with earlier studies conducted in this population as well as those carried out in other populations.

**Methods:**

The study group consisted of 456 boys and 514 girls aged 14-18 years living in Cracow chosen from randomly selected secondary schools. Weight, height, waist, and hip circumference (WC, HC) as well as triceps, biceps, subscapular, and suprailiac skinfold thickness (SFT) were measured. Body mass index (BMI), waist-to-hip ratio (WHR), waist-to-height ratio (WHtR), subscapular/triceps skinfold ratio (STR), and percentage body fat were computed. The prevalence of overweight and obesity based on Polish children growth reference were calculated and age-dependent and gender-specific smoothed percentile curves for BMI and ROC curves were generated.

**Results:**

Weight, height, WC, HC (up 16yr), WHtR (up 15yr), and WHR were considerably higher in males than females. Weight, height, and HC increased with age; WHtR remained the same. The prevalence of overweight and obesity were 10.2% (boys 10.3%; girls 10.1%) and 4.2% (boys 5.3%; girls 3.3%). ROC analysis revealed that WHtR was the best tool for detection of obesity (AUC of 0.982±0.007) in males, whereas the sum of four SFTs (AUC: 0.968±0.011) and WHtR (AUC: 0.963±0.012) were the best predictors of obesity in females.

**Conclusions:**

The level of adiposity in Cracow adolescents increased during the last decade. However, it is still lower than in other well-developed societies struggling with obesity epidemics.

## Introduction

The increasing adiposity among children and adolescents is becoming a global health concern both in developed and developing countries [1–3]. In 2010, an estimated 43 million children worldwide were considered to be overweight and obese (35 million in developing countries) [4]. The global prevalence of overweight and obesity among children increased from 4.2% in 1990 to 6.7% in 2010 and is expected to reach 9.1% by 2020 [4]. Childhood obesity is a serious risk factor for the development of obesity in adulthood which impacts the overall state of health of the individual by increasing the risk of developing diabetes mellitus, hypertension, coronary artery diseases, and metabolic syndrome[5,6]. Furthermore, increased adiposity levels have been linked to depression indicating that psychological morbidities are also important consequences [7]. Therefore, it is imperative to monitor the body parameters of children and adolescents in order to prevent the accumulation of excess body fat and its associated morbidities. Body fat can be measured with various tools and indicators. These indicators can be used to describe several types of obesity however carry different limitations on the sensitivity and reliability of the data obtained. Thus, care must be taken in the selection of an appropriate tool to be used for the particular population of interest. The most commonly utilized tool is the body mass index (BMI) which is used by the WHO as the standard for recording obesity statistics and constructing growth curves for school-aged children and adolescents [8]. Despite the many advantages of this index, the BMI also has limitations. It does not take into consideration many factors such as size of muscle tissue [9], bone density, varying proportions of fat, cartilage, bone tissue, and body water. A highly specific and sensitive anthropometric marker of central obesity in children and adolescents is waist circumference (WC). Conversely, the waist to hip ratio (WHR) is not an accurate indicator of abdominal obesity in youth because of the weak correlation with central adiposity [10]. Measurements of skinfold thickness (SFT) can be used as a specific index for obesity due to its high sensitivity. Because of the latter, they are recommended to observe obesity in children and adolescents [11] and they provide an indirect estimate of total body fat that can be used as a screening tool. According to Nooyens et al. [12], the best predictor of adult body fatness in males is subscapular SFT and in females biceps SFT. Both of these single skinfold thickness measures were better predictors than the sum of the four SFT. Moreover, truncal obesity can effectively be assessed using the subscapular/triceps skinfold ratio (STR) [13]. Therefore, obesity in adulthood is better predicted by adolescent skinfold thickness than by adolescent BMI. Thus, it is important to test out how other indices, such as skinfold thickness, might be applied to the Polish population. In the present study, we utilized the BMI cut-offs that have been found to be appropriate in describing the Polish population by the OLAF study [14] to define obesity status. We further sought which indices may be just as effective in comparison while offering some of the advantages described above to provide the most complete anthropometric description of the adiposity among Polish youth.

The goal of the present research was to predict and determine the level of adiposity and obesity among Polish adolescents. Collected data can be used to document and describe changes in obesity over time in Polish youth. Monitoring anthropometric changes in the population can be pivotal in preventing future public health issues like obesity. Such research can also help elucidate the important roles of education and physical activity in youth in the prevention of negative health outcomes related to adiposity. Regular anthropometric assessments of the population may provide key information that would allow heath institutions to determine the points at which they should begin mounting interventions.

## Material and Methods

### Study population

Data was collected during the 2012/2013 school year from 8 secondary schools dispersed in various districts within the city of Cracow. The study population included 456 male and 514 female students, aged 14 to 18.

The study was approved by the Bioethical Committee of the Jagiellonian University Medical College, Cracow (KBET/240/B/2013). The subject of the project was explained to local school authorities and their written approval was obtained before seeking permission from individual students and their parents to participate in the voluntary measurements. Permissions were collected in the form of written informed consent obtained from all children who participated in the study and their parents or guardians. All students from the randomly selected classes were invited to participate. The exclusion criteria were: (1) Parent or student refusal. (2) Pre-existing medical conditions such as severe genetic diseases (e.g. Down’s syndrome or Marfan’s syndrome); severe hormonal abnormalities (e.g. Cushing’s disease/syndrome); diseases leading to swelling of subcutaneous tissues; diseases leading to muscle wasting; and metabolic bone diseases. (3) Taking medication that might affect body indices. Initially 1400 students and their legal guardians were approached to participate and of these roughly 70% agreed to participate (981 students). A total of five students were excluded due to the presence of the previously listed medical conditions.

### Measurement

All study personnel was required to pass a three day training course guided by doctors and specialists with anthropometric training before being allowed to work with study participants. In order to successfully finish the course, the medical students had to complete at least 20 sets of measurements with each differing less than 5% from those made on the same individuals by the specialists.

To minimize both human and instrument errors, all sets of measurements were taken independently by two well–trained medical students with separate equipment under the supervision of medical doctors. Data was then averaged to the nearest 0.1cm and 0.1kg. If the measurements collected by the two students differed by more than 10% then they were not included in the database. Data from six participants was omitted on account of such discrepancies. Weight and height was measured using an electronic scale with height meter (Radwag model WPT 60/150 OW, Poland, accurate to 0.1cm and 0.1kg). Children were asked not to wear shoes or any heavy clothing during the measurements. Participants were instructed to stand straight on the scale with their feet together on the horizontal plane and both hands hanging freely by their sides. BMI was calculated as weight divided by height squared (kg/m^2^).

Skinfolds thickness (SFT) was measured to the nearest half millimeter on the right side of the body using a Gima’s Skinfold Caliper FAT-1. Skinfolds were measured by grabbing them between the index finger and the thumb. The skin was then gently pinched with the caliper to measure the skinfold thickness in millimeters. The mean of two measurements was taken [9,15]. Triceps skinfolds were measured on the back of the arm with the elbow extended, exactly halfway between the acromion and the proximal end of the olecranon. Biceps skinfolds were measured at the mid-length of the biceps. Subscapular skinfolds were measured 1cm below the inferior angle of the scapula. The angle between the long axis of the skinfold and the long axis of the body was equal to 45° in accordance to WHO standards. Suprailiac skinfolds were measured about 2.0cm above the iliac crest and 2.0cm towards the medial line. The waist circumference and the hip circumference (HC) were measured with an inextensible measuring tape. WC was measured horizontal to the floor halfway between the iliac crest and the lowest rib at the end of each breath. The hip circumference was measured around the widest portion of the participant’s buttocks according to WHO guidelines [16]. The waist to height ratio (WHtR) was obtained by dividing WC by height. Subscapular/triceps skinfold ratio was computed as the subscapular SFT divided by the triceps SFT. Slaughter's equation was used to calculate percentage Body Fat (%BF) [17].

The classification of overweight and obesity status was determined according to the most recent Polish children growth reference 2010 (OLAF study) [14]. Each child’s BMI was compared with reference values by sex and age. Overweight status was defined by a BMI higher than the 85th percentile but lower or equal to the 95th percentile in relation to gender and age. Obesity was established by a BMI greater than the 95th percentile. The waist to hip ratio for adolescents identified as obese according to the foregoing criteria was calculated as WC divided by HC.

### Statistical analysis

Calculations were performed using Statistica 10.0 and Excel 2010 for Windows. Data was presented as the mean and standard deviation (SD). For BMI values percentiles curves were developed using the LMS method [18] and divided by sex and age. BMI outcomes were compared with 2010 growth references for Polish children and adolescents (OLAF study) [14]. To compare results between genders, t-tests and nonparametric Mann-Whitney U tests were conducted. P-values less than 0.05 were considered to be statistically significant. Correlation coefficients were calculated to measure statistical dependence between anthropometric indices and age. Based on the OLAF study BMI cut-off values, we determined which subjects were obese. Receiver operating characteristic (ROC) curves were then constructed and assessed to see which other indices, such as SFT, would be as effective as the BMI in describing the obesity status of Polish youth.

## Results

A total of 970 adolescents were measured. The study group consisted of 456 boys and 514 girls with a mean age of 15.9 ± 1.5 years.

Table 1 shows the means of the anthropometric variables collected by age and gender. Weight, height and HC increased with age and were significantly higher in males (respectively: r = 0.5; r = 0.54; r = 0.38. All p<0.001) than females (respectively: r = 0.3; r = 0.32; r = 0.22. All p<0.001). WHtR remained constant in boys and decreased in girls (r = -0.2, p<0.001). WC increased with age only in boys (r = 0.38; p<0.001). From age 16 to 18 HC was higher in males. Similarly, BMI (ages 17 and 18) and WHtR (ages 15, 17, and 18) values were also greater in males. No WHR results are reported for 16 year old girls because of the absence of obese adolescents in this group.

**Table 1:** Mean values (±SD) of body measurements in male and female Polish adolescents aged 14–18.

Age (yr)	n	Weight (kg)	Height (cm)	WC (cm)	HC (cm)	BMI (kg/m2)	WHtR	WHR
**Boys**								
14	121	55.5 ± 11.1*	165.7 ± 8.5^b^	73.6 ± 9.7*	87.9 ± 8.4	20.1 ± 3.0	0.44 ± 0.05	0.91 ± 0.05
15	129	60.8 ± 12.6*	172.1 ± 6.3*	76.0 ± 9.9*	91.0 ± 7.8	20.4 ± 3.5	0.44 ± 0.05*	0.93 ± 0.05
16	68	67.3 ± 9.3*	177.0 ± 7.2^b^	77.9 ± 7.5*	94.2 ± 5.7*	21.5 ± 2.7	0.44 ± 0.04	0.93 ± 0.02
17	54	70.0 ± 10.5*	178.1 ± 6.5*	76.7 ± 8.0*	94.8 ± 6.6^b^	22.0 ± 2.9*	0.43 ± 0.04*	0.88 ± 0.06
18	84	70.2 ± 11.6*	177.7 ± 5.5^b^	76.3 ± 8.1*	95.2 ± 6.9*	22.2 ± 3.6*	0.43 ± 0.05*	0.86 ± 0.08
**Total**	**456**	63.2 ± 12.7*	172.9 ± 8.5*	75.8 ± 9.1*	91.9 ± 7.9*	**21.0 ± 3.3**	0.44 ± 0.05*	0.91 ± 0.06*
**Girls**								
14	101	51.2 ± 8.9	160.0 ± 6.5	69.7 ± 7.9	86.6 ± 7.9	20.0 ± 2.9	0.44 ± 0.05	0.84 ± 0.06
15	101	55.2 ± 11.0	164.8 ± 6.6	71.0 ± 9.7	89.6 ± 8.2	20.2 ± 3.1	0.43 ± 0.05	0.89 ± 0.05
16	80	56.0 ± 6.6	164.0 ± 5.2	70.3 ± 5.9	88.2 ± 6.1	20.8 ± 2.4	0.43 ± 0.04	-
17	82	56.9 ± 8.8	164.7 ± 6.7	68.0 ± 6.7	89.5 ± 7.2	20.9 ± 2.5	0.41 ± 0.04	0.80 ± 0.05
18	150	58.6 ± 8.3	166.9 ± 5.5	69.1 ± 6.8	91.7 ± 7.8	21.0 ± 2.6	0.41 ± 0.04	0.82 ± 0.07
**Total**	**514**	**55.8 ± 9.2**	**164.3 ± 6.5**	**69.6 ± 7.5**	**89.4 ± 7.8**	**20.6 ± 2.8**	**0.42 ± 0.05**	**0.84 ± 0.06**

* p<0.05 if significant sex difference—nonparametric.

^b^ p<0.05 if significant sex difference—parametric.

Skinfolds measurements values and BF% are presented in Table 2. Biceps, triceps, and subscapular SFT were considerably higher in females than males. Biceps SFT and triceps SFT decreased in males with age while the latter increased with age in females. Furthermore, suprailiac SFT increased in females with age and was significantly higher than in males (ages 17 and 18). STR was significantly higher in males except at age 14 and increased with age (r = 0.3; p<0.001). In contrast, the latter ratio decreased with age in females (r = -0.19; p<0.001). Total mean BF% in females was statistically higher than in males. Moreover, between age 14 and 18 BF% increased by 1.1% in girls and decreased by 3.8% in boys.

**Table 2:** Mean values (±SD) of skinfold thickness and percentage body fat in male and female Polish adolescents aged 14–18.

Age (yr)	n	Biceps	Triceps	Subscapular	Suprailiac	∑ Skinfolds	Subscapular/triceps	Body Fat %
**Boys**								
14	121	7.6 ± 5.2	13.1 ± 7.8	10.6 ± 7.3	10.9 ± 8.1	42.3 ± 26.7	0.84 ± 0.34	20.0 ± 11.6
15	129	6.5 ± 4.6	11.1 ± 6.2	9.9 ± 7.0	9.8 ± 7.4	37.2 ± 23.4	0.90 ± 0.31*	16.0 ± 10.7
16	68	6.8 ± 5.2	12.1 ± 6.3	10.2 ± 5.5	9.7 ± 7.0	38.9 ± 21.1	0.92 ± 0.39*	17.2 ± 9.2
17	54	5.8 ± 3.9	10.3 ± 4.6	9.9 ± 4.6	10.6 ± 7.1	36.6 ± 17.8	1.03 ± 0.37*	15.4 ± 7.2
18	84	5.3 ± 3.3	10.4 ± 6.5	10.7 ± 5.4	11.2 ± 7.5	37.6 ± 20.9	1.17 ± 0.44*	16.2 ± 9.6
**Total**	**456**	**6.5 ± 4.6**	**11.6 ± 6.7**	**10.3 ± 6.3**	**10.4 ± 7.5**	**38.8 ± 23.0**	0.95 ± 0.38*	**17.2 ± 10.3**
**Girls**								
14	101	9.8 ± 6.8^b^	16.0 ± 8.2*	12.5 ± 7.4*	11.9 ± 8.3	50.2 ± 27.8*	0.83 ± 0.35	23.7 ± 9.3*
15	101	9.7 ± 6.0*	16.1 ± 7.8*	11.8 ± 6.8*	10.9 ± 7.3	48.4 ± 25.6*	0.78 ± 0.29	23.5 ± 8.7*
16	80	9.2 ± 4.8*	17.1 ± 6.9*	12.6 ± 6.1*	11.5 ± 7.2	50.4 ± 21.3*	0.79 ± 0.30	24.8 ± 7.4*
17	82	8.9 ± 4.2*	17.3 ± 5.4*	11.6 ± 4.9*	12.7 ± 6.3*	50.5 ± 17.4*	0.70 ± 0.26	24.6 ± 5.7*
18	150	9.2 ± 4.9*	17.6 ± 5.7*	11.6 ± 4.9*	13.7 ± 6.5*	52.1 ± 18.0*	0.69 ± 0.29	24.8 ± 5.8*
**Total**	**514**	9.4 ± 5.4*	16.9 ± 6.9*	12.0 ± 6.0*	12.3 ± 7.2*	50.5 ± 22.2*	**0.75 ± 0.30**	24.3 ± 7.4*

* p<0,05 if significant sex difference.

^b^ p<0,05 if significant sex difference—parametric.

**Fig 1:**
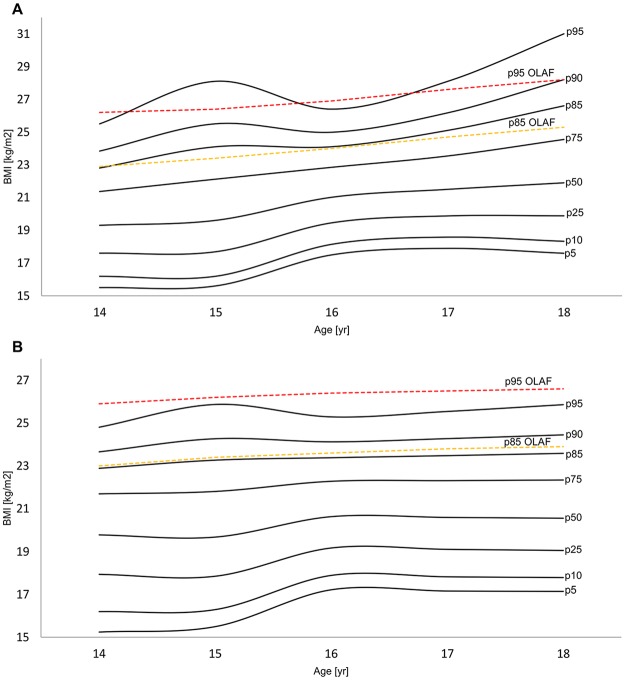
Smoothed percentiles of BMI for Polish adolescents with OLAF study [14] cutoffs (Polish children growth reference 2010). Boys (A), Girls (B).

Age-dependent and gender-specific smoothed BMI percentile curves and their particular values are shown in Fig. 1 and Table 3. According to the OLAF study [14] BMI cut-off norms there were 71 (15.6%) males and 69 (13.4%) females considered overweight (including obesity), which represents 14.4% of all participants. Obese status was determined in 24 (5.3%) boys and 17 (3.3%) girls. These values are presented in the Table 4.

**Table 3:** Percentile values of body mass index (BMI) in male and female Polish adolescents aged 14–18 with corresponding values from the OLAF study [14] for the 85^th^ and 95^th^ percentiles.

	Age (yr)	Percentiles									
		5th	10th	25th	50th (Me)	75th	85th		90th	95th	
**Boys**								OLAF			OLAF
BMI	**14**	15.5	16.2	17.6	19.3	21.4	22.8	22.9	23.8	25.5	26.2
	**15**	15.6	16.2	17.7	19.6	22.1	24.1	23.4	25.5	28.1	26.4
	**16**	17.5	18.1	19.5	21.0	22.8	24.1	24.0	25.0	26.4	26.9
	**17**	17.9	18.6	19.9	21.5	23.5	25.1	24.7	26.2	28.1	27.6
	**18**	17.6	18.3	19.9	21.9	24.6	26.6	25.3	28.2	31.0	28.2
**Girls**								OLAF			OLAF
BMI	**14**	15.2	16.2	17.9	19.8	21.7	22.9	23.0	23.7	24.8	25.9
	**15**	15.5	16.3	17.8	19.7	21.8	23.3	23.4	24.3	25.9	26.2
	**16**	17.2	17.9	19.2	20.6	22.3	23.4	23.6	24.1	25.3	26.4
	**17**	17.2	17.8	19.1	20.6	22.3	23.5	23.8	24.3	25.5	26.5
	**18**	17.1	17.8	19.0	20.6	22.3	23.6	23.9	24.5	25.9	26.6

**Table 4:** The prevalence of overweight and obesity in Cracovian boys and girls aged 14–18 based on Polish children growth reference 2010 (OLAF study) [14].

gender	n	BMI >p85	BMI p85-95	BMI >p95
			Overweight	Obesity
**Boys**	456	71	47	24
		15.6%	10.3%	5.3%
**Girls**	514	69	52	17
		13.4%	10.1%	3.3%
**Total**	970	140	99	41
		14.4%	10.2%	4.2%

**Fig 2:**
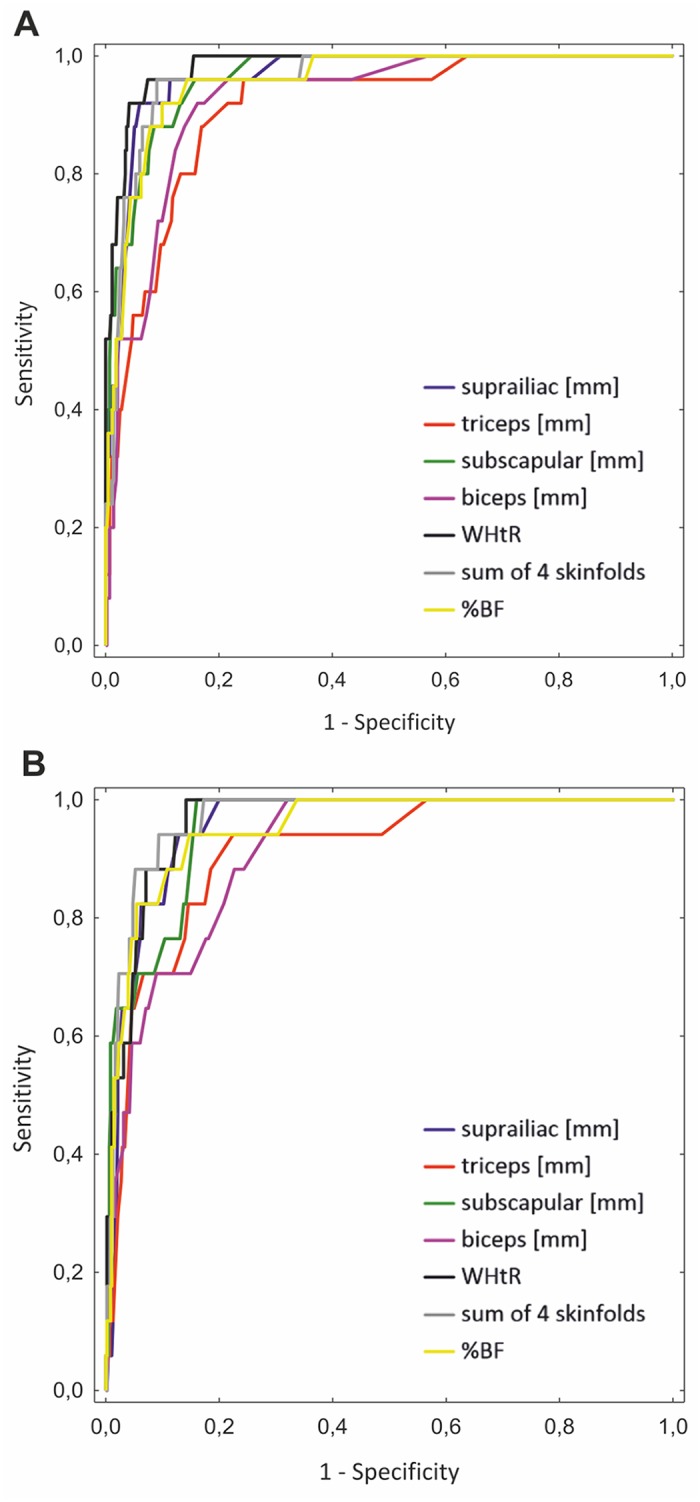
ROC curves for predicting obesity according to the growth reference charts from the 2010 OLAF study [14] obtained for waist-to-height ratio (WHtR), percentage body fat (%BF), biceps, triceps, subscapular, suprailiac skinfolds and their sum. Boys (A), Girls (B).

The ROC curves of measured and computed indicators for predicting obesity on the basis of OLAF study BMI cut-offs are shown in Fig. 2. Graphs reveal that the best obesity predictor for males was WHtR (area under the curve [AUC]: 0.982 ± 0.007; 95%CI 0.967–0.996), followed by the suprailiac SFT (AUC: 0.965 ± 0.013; 95%CI 0.941–0.990), and subscapular SFT (AUC: 0.963 ± 0.013; 95%CI 0.939–0.987). Whereas, for females the sum of 4 SFTs (AUC: 0.968 ± 0.011; 95%CI 0.946–0.990), followed by the WHtR (AUC: 0.963 ± 0.012; 95%CI 0.940–0.985), and then the suprailiac SFT (AUC: 0.955 ± 0.013; 95%CI 0.929–0.981) values were the best predictors of obesity.

## Discussion

The current study presents mean values for BMI, WHR, WHtR, biceps, triceps, subscapular and suprailiac SFT, STR, as well as age and gender-specific percentile curves for BMI in a sample of 970 Polish adolescents living in Cracow aged 14–18 years. Cracow is a city of uniform ethnicity and our study includes ~2.5% of considered population [19]. Thus, the sample of children evaluated in the present manuscript is representative of Cracow youth.

The aim of this study was to determine the level of adiposity and obesity in adolescents using several anthropometric indicators and compare the results with earlier studies conducted on this population as well as those carried out in other populations. BMI cut-offs from the OLAF study were used to establish overweight and obesity status instead of those outlined by the IOTF [19]. Previous studies have found that the IOTF values resulted in an underestimation of obesity in Polish youth thus using Polish cut-off values was more appropriate [20,21].

The present results are considerably different from the results of studies conducted in Cracow between 1980 and 1992 [22] as well as in 2000 [23]. Furthermore, the results also vary from investigations conducted three years ago in various Polish adolescent populations [5,24,25]. The present SFT measurements, especially the triceps SFTs, are considerably higher than those reported between 1980 and 1992 [22]. Comparing the mean WC values obtained in this study with the results obtained from Cracow adolescents in 2000 [23], the current results are much higher in all age groups. Interestingly, the present mean values of HC are higher for males of all ages and lower for females except at age 18 where the female values are slightly higher than previous studies [23]. This study showed higher percentile and median values for each of the triceps, subscapular and suprailiac SFT for males (14–18 years of age) and for females (14–18 years of age), except the subscapular STF for females (17–18 years of age) which was slightly lower than previously reported in Cracow studies [23]. Furthermore, a similar trend can be observed in the comparison with other Polish populations [25], except for the suprailiac SFT in females in all age groups where the mean values of the present study were lower. Equally the mean BMI values of the present study population were slightly higher across all age groups than those obtained previously. These differences were slightly higher in males than in females but were not statistically significant. In summary, most of the indicators of adiposity are higher in the current investigation in relation to studies conducted several years ago, which may hint at the progressive development of an epidemic of obesity in Polish society.

Comparison of our results with the global data collected over the past 13 years showed many differences across the parameters measured. BMI for Cracow girls of all age groups was higher than in girls from Hong Kong [26], China [27] and Nigeria [3] (except females 18 years of age) and lower than girls from Germany [28], America [29], Bahrain[30], Bolivia [31], Cypria [32], Bulgaria [33], Spain [13], and South Korea [34]. For Cracow boys BMI was higher than those of Nigerian [3], China [27], Bahrain [30], Hong Kong (except 15 years of ages) [26] boys and lower than American [29], Spanish [13], Cypriot [32], and Bulgarian [33] boys. In most cases these differences were slight (except America [29]) and age dependent.

In our study, we reveal that 10.3% of males and 10.1% of females are considered overweight (BMI p85-95), which represents 10.2% of all participants. Meanwhile 5.3% of boys and 3.3% of girls were classified as obese (BMI >p95) which represents 4.2% of all participants. Aggregate percentages of obesity and overweight status (BMI >p85) were 15.6% males and 13.4% females (14.4% of all participants). It is difficult to compare the findings of this study with many previously published studies because of the differences in criteria for overweight and obese status regarding both the standard used and the age range. Therefore, comparisons between results is focused on those studies whose methodology and study population are quite similar to those of the present study. Using the cut-off points recommended by IOTF, Lobstein & Frelut [35] present the percentage of overweight (overweight includes obese) adolescents aged around 14–17 years in various countries: Slovakia (8%), Russia (9%), Czech Republic (9%), The Netherlands (11%), Poland (12%), Germany (13%), Denmark (17%), Bulgaria (17%), Croatia-Zagreb (20%), UK (21%), Spain (21%), Greece-Thessaloniki (22%), and Cyprus (23%). In comparison to the above mentioned countries, the Polish youth studied in the current study seems to fall near the middle of the 8–23% range presented (14.4%). Comparing the current study’s results to those of Arab countries [36] reveals a troubling prevalence of excessive body weight as shown by the high combined percentage of overweight and obese individuals in Kuwait (60.4%; 41.4%), UAE-Sharjah city (38.9%; 20.2%) and Jordan-Amman (31.8%; 22.1%) in males and females respectively. Comparison of our %BF results with the German [28] data reveals higher percentages of body fat in adolescents from Cracow (14–18 years of ages).

There were non-significant differences between the WC values of Cracowian youth and those of Bulgarian [33], Spanish [13], and Cypriot [32] adolescents. However there were considerably lower WC values for Hong Kong (boys and girls) [26] and Nigerian boys [3] as compared to those of Cracow youth. Mean HC values in our study population were slightly lower than German (14–18 years of ages) [28] and Spanish (14, 15, 17 years of ages) [13] youth and substantially higher than Nigerian (14–18 years of ages) [3] boys and girls of all age groups. WHR was very similar for adolescents in both sexes and across all age groups in Cracow, Nigeria [3], Germany [28] and Spain [13] except in Nigerian girls (14–18 years of ages) where it was higher.

Mean biceps and subscapular STF values in Cracow girls (14–18 years of age) were much lower than those of Bahrain [30] adolescents while for boys they were slightly higher or similar. These indicators were considerably higher in the present Polish adolescents when compared to Nigeria [3] (except for 18 year old girls) and were similar to Spanish data [13]. There were no significant differences between mean STR values for German [28] and Cracow adolescents. There was a higher accumulation of fat in the area of the shoulder girdle in American [29] adolescents than in the present Cracow study population. The observed differences in the level of adiposity may be due to ethnic and genetic differences between the studied populations, geographic and socio-economic conditions, variations in food composition, patterns of food intake, and physical activity.

Based on ROC analysis, WHtR was the best tool for detection of obesity (AUC of 0.982 ± 0.007) in Cracow male adolescents, whereas the sum of 4 SFTs (AUC: 0.968 ± 0.011) was the best predictor of obesity in Cracow girls aged 14–18 years. Chrzanowska and colleagues suggest that puberty related fat distribution changes lead to a contrast between trunk and extremity fatness in boys while smaller changes in girls lead to a more even distribution of fatness [22]. Such a finding might explain why in the present study the sum of the 4 SFTs, which represents a mix of truncal (subscapular and suprailiac SFT) and extremity (triceps and biceps SFT) values, is a good predictor of obesity in girls but is not as useful in boys. Equally good indicators were suprailiac STF and subscapular STF for boys and WHtR and suprailiac STF for girls. Earlier studies on German [28] and Portuguese [37] adolescent revealed similar relationships. The WHtR was the best predictor for abdominal obesity in both German boys (AUC: 0.974 ± 0.004) and girls (AUC: 0.986 ± 0.003). It was followed by %BF, subscapular SFT, and the sum of the SFTs in boys and by WHR, the sum of the SFTs, and %BF in girls [28]. In 12–15 year old Portuguese adolescents the AUC for triceps SFT ranged from 0.94 ± 0.045 to 0.86 ± 0.087 in boys and from 0.94 ± 0.034 to 0.95 ± 0.036 in girls. Measurements of triceps SFT was assessed as the best screening tool for obesity in boys and girls at this age [37].

Based on our ROC analysis and the comparisons presented above, we conclude that WHtR and SFT measurements should be used as the preferred screening tools. SFT is easy to perform and predicts later adult body fatness better than adolescent BMI does. Stomfai et al. [38] found that intra-observer reliability for SFT measurements (triceps, subscapular, biceps, suprailiac) was 97.7% and inter-observer technical error of measurement for skinfold thicknesses was between 0.13 and 0.97 mm. Furthermore, since %BF can be calculated on the basis of SFT measurements these measurements seem to be very useful screening tools along with WHtR measures.

The main limitation of this study is that about 30% of parents refused to consent to the participation of their children in the measurements. Reasons for non-participation were not recorded so it is not possible to determine whether non-participation had a significant influence on the results of the study. For example, it is possible that parents of children who were overweight didn’t want their child to participate hence affecting the prevalence of excess adiposity recorded. Further limitations related to the cross-sectional design of the study include that the physical activity, nutritional habits, lifestyle choices, and socio-economic characteristics of the study population were not recorded. Comparisons of data should be interpreted cautiously considering important methodological limitations such as the anatomical measurement site, the time of year that the data was collected, the accuracy of the measuring equipment, the method used for the derivation of the percentile curves, and overlapping populations and age groups.

The strengths of our study include the fact that we were the first to present biceps SFT mean values for Polish adolescents. According to the authors' best knowledge, this study is the first to show the mean values of STR for such a large population of Polish adolescents.

## Conclusions

The level of adiposity in Cracow adolescents has increased over the last 10 years yet remains lower than in other well-developed societies faced with an obesity epidemic. The present investigation can be used as baseline data for a long-term observation of body fat and obesity trends among Cracow (Polish) adolescents and to compare anthropometric indicators to other populations. We strongly recommend performing similar studies on adolescents from other populations around the world. To properly track changes in adiposity level, studies should be performed every decade. We conclude that SFT measurements should be used as the preferred screening tool because they are simple to perform and are better predictors of adult body fatness than adolescent BMI. Based on ROC analysis, WHtR was the best tool for detection of obesity in males whereas in females the sum of 4 SFTs and WHtR were the best tools.
